# 30 years of polio campaigns in Ethiopia, India and Nigeria: the impacts of campaign design on vaccine hesitancy and health worker motivation

**DOI:** 10.1136/bmjgh-2021-006002

**Published:** 2021-08-03

**Authors:** Abigail H Neel, Svea Closser, Catherine Villanueva, Piyusha Majumdar, S D Gupta, Daniel Krugman, Oluwaseun Oladapo Akinyemi, Wakgari Deressa, Anna Kalbarczyk, Olakunle Alonge

**Affiliations:** 1 International Health, Johns Hopkins University Bloomberg School of Public Health, Baltimore, Maryland, USA; 2 SDG School of Public Health, Indian Institute of Health Management Research, Jaipur, Rajasthan, India; 3 Anthropology, Middlebury College, Middlebury, Vermont, USA; 4 Health Policy and Management, University of Ibadan College of Medicine, Ibadan, Nigeria; 5 Preventive Medicine, Addis Ababa University, Addis Ababa, Ethiopia

**Keywords:** health systems, poliomyelitis, vaccines

## Abstract

**Introduction:**

The debate over the impact of vertical programmes, including mass vaccination, on health systems is long-standing and often polarised. Studies have assessed the effects of a given vertical health programme on a health system separately* *from the goals of the vertical programme itself. Further, these health system effects are often categorised as either positive or negative. Yet health systems are in fact complex, dynamic and tightly linked. Relationships between elements of the system determine programme and system-level outcomes over time.

**Methods:**

We constructed a causal loop diagram of the interactions between mass polio vaccination campaigns and government health systems in Ethiopia, India and Nigeria, working inductively from two qualitative datasets. The first dataset was 175 interviews conducted with policymakers, officials and frontline staff in these countries in 2011–2012. The second was 101 interviews conducted with similar groups in 2019, focusing on lessons learnt from polio eradication.

**Results:**

Pursuing high coverage in polio campaigns, without considering the dynamic impacts of campaigns on health systems, *cost* campaign coverage gains over time in weaker health systems with many campaigns. Over time, the systems effects of frequent campaigns, delivered through parallel structures, led to a loss of frontline worker motivation, and an increase in vaccine hesitancy in recipient populations. Co-delivery of interventions helped to mitigate these negative effects. In stronger health systems with fewer campaigns, these issues did not arise.

**Conclusion:**

It benefits vertical programmes to reduce the construction of parallel systems and pursue co-delivery of interventions where possible, and to consider the workflow of frontline staff. Ultimately, for health campaign designs to be effective, they must make sense for those delivering and receiving campaign interventions, and must take into account the complex, adaptive nature of the health systems in which they operate.

**Key questions**

**What is already known?**

- Vertical programmes including mass vaccination campaigns have a range of impacts, both positive and negative, on health systems.
- Health systems as are complex, dynamic systems that change over time.

**What are the new findings?**

- Applying a complex adaptive systems (CAS) approach to the relationships between polio campaigns and broader health systems highlights some implementation pathways that are less apparent in more traditional, static approaches to health systems analysis.
- CAS analysis highlights the interconnectedness of systems dynamics, for example, frontline health worker motivation and community trust.
- Both vaccine hesitancy and worker fatigue were driven by the interaction of mass vaccination campaigns with weaker health systems; these dynamics took time to develop.

**What do the new findings imply?**

- Mass vaccination campaigns, including COVID-19 vaccine campaigns, should plan for both worker fatigue and vaccine hesitancy over the long haul if multiple and frequent mass campaigns are not integrated with broader health system activities.
- Codelivering other interventions in mass vaccination campaigns is a key way to minimise negative dynamics when relying on parallel structures is unavoidable.

## Introduction

Systematic research exploring the impacts of mass vaccination campaigns on health systems began in the 1990s, with a study led by Carl Taylor examining the impact of polio campaigns on health systems in the Americas.^1^ This report concluded that the polio programme had largely positive though varying effects on health systems, yet warned readers against extrapolating the findings to other regions. This work established two modes of thought that shaped future research in profound ways. First, it evaluated the impacts of vertical programmes on health systems *separately* from the question of whether they achieved their own internal goals. The Taylor Commission did not evaluate the efficacy of the polio programme itself; the programme had already succeeded at eliminating polio in the region.

Second, the report evaluated the impacts of vertical programmes on health systems *in a binary manner*, with impacts separated by health system component, and categorised as positive or negative. For example, the polio programme had a significant positive effect on management strategies of the broader health system; it had a modest positive effect on interagency and intersectoral collaboration; and a significant negative effect on availability of scarce resources—‘vaccination campaigns were resented because everything else had to be interrupted in order to carry them out’ (p61). The Taylor Commission was fully aware that these impacts were complex—in some categories, both positive *and* negative effects were listed—but the framework for presenting these effects was relatively simple.

In the intervening 25 years, there has been a great deal of sophisticated research evaluating the impacts of mass campaigns on health systems within a variety of settings.^2–10^ This body of work has described the push and pull between campaign-based and routine delivery strategies,^10^ and highlighted the inherent tensions in trying to leverage eradication initiatives for health systems strengthening.^11^ This research has largely hewed to the methodological precedents of separating the impacts of campaigns by health system component, and considering impacts in a binary manner.^12^ These methodologies were adopted because they provided a straightforward way of examining complex systems.

Yet the simplicity of the dominant frameworks limited their utility, as the researchers involved were well aware. In 2014, the authors of three studies on mass vaccination campaigns argued that better frameworks for such studies were needed. ‘Health systems are complex,’ they wrote, ‘like a living organism, they are dynamic, with interacting components—at various geographical levels—that lead to adaptation and to the emergence of new dynamics’.^12^ Subsystems such as routine immunisation (RI) are also complex and dynamic.^13^

While systems thinking approaches have been increasingly applied to complex phenomena in global health,^14 15^ they remain underutilised. With the advent of global COVID-19 vaccination campaigns, there is renewed attention both on how to maximise an individual campaign’s effectiveness^16^ as well as plan a campaign to benefit the health system.^17 18^ This work continues to consider the *health systems* impacts of campaigns separately from the success of the campaign itself.

To move beyond these prevailing ways of thinking requires describing health systems as they are—complex, dynamic and tightly linked—and making explicit the relationships between elements of the system which affect system outputs and outcomes over time. We propose a complex adaptative systems (CAS) lens. CAS thinking includes the notion of ‘path dependency,’ that processes with similar inputs and governing mechanisms may lead to very different outcomes,^19^ and emphasises the unintended, even paradoxical, effects that can occur within these complex systems.^19 20^

We use CAS to explore the relationships between polio campaigns and health systems in Ethiopia, India and Nigeria. Unlike previous analyses, which have focused on the polio programme’s impacts on health systems, our analysis focuses on how *interactions* between the polio programme and the health system influence two key outcomes, frontline health worker (FLHW) motivation and vaccine hesitancy. (We take the definition of vaccine hesitancy as ‘delay in acceptance or refusal of vaccination despite availability of vaccination services’^21^; hesitancy has multifactorial determinants, including issues beyond the vaccine itself, and varies by population and context^22–24^). These two factors, in turn, affect campaign coverage in the polio programme itself.

## Methods

### Data sources

This paper describes an analysis of qualitative data sourced from two studies. The Polio Eradication Impacts Study, conducted in 2011–2012, explored the relationship between the Global Polio Eradication Initiative (GPEI), RI, and primary health care (PHC) in seven study sites using a multimethods approach.^3^ The Synthesis and Translation of Research and Innovations from Polio Eradication (STRIPE) study, conducted in 2019, mapped explicit and tacit knowledge from polio eradication at the global level and in seven countries.^25^ The STRIPE study focused on implementers directly involved in polio eradication for at least 12 months,^26^ whereas the Impacts study included staff working on polio, RI, and PHC more broadly. Both studies included frontline, district, and national level respondents; the STRIPE study also included global actors. Oral consent was received from all respondents and confidentiality maintained.

We constructed a causal loop diagram (CLD) from analysis of semistructured interviews drawn from both studies in three countries: Ethiopia, India, and Nigeria (table 1), as well as interviews with global policy-makers (n=17) conducted as part of STRIPE. We also conducted a review of the literature on polio eradication in those countries.

**Table 1:** Semistructured Interviews conducted as part of the Polio Eradication Impacts^3^ and Synthesis and Translation of Research and Innovations from Polio Eradication (STRIPE)^26^ studies and analysed in this paper

	Ethiopia	India	Nigeria
Polio Eradication Impacts Study (2011–2012)			
National level officials	5	1	3
District level officials	7	15	5
Frontline* workers	36	59	22
Total	55	85	35
STRIPE Study (2019)			
National level officials	7	11	10
District level officials	17	10	13
Frontline* workers	6	4	6
Total	30	25	29

*Frontline health workers include community health workers, health extension workers, or vaccination staff (ie, those who are directly delivering oral polio vaccine).

Our three focus countries have had substantial polio campaign activity over many years, and encompass wide differences in context, number, and type of campaigns. (Throughout this paper, when we refer to ‘campaigns’ we mean oral polio vaccine (OPV) vaccinations delivered separately from RI as part of Supplementary Immunization Activities). They thus provide a rich set of information for understanding the complex interactions between campaigns, health systems and communities.

### Study countries

*India* is home to the largest, most extensive, and most diverse polio programme in the world. While polio was eliminated quickly in South India, the northern states of Uttar Pradesh and Bihar, which had lower coverage of key health interventions like immunisation, were targeted with an intense schedule of campaigns, as many as 10–12 per year in some areas in the late 2000s. Polio was finally eliminated from the country in 2010 through an intense government-financed focus on repeated campaigns, along with some additional health systems strengthening measures in key areas of Uttar Pradesh and Bihar.^3^ Since that time, India has kept immunity high enough to prevent both imported cases and outbreaks of circulating vaccine derived polio (a form of genetically mutated vaccine-virus which can cause paralysis^27^), while also transitioning some key polio functions like surveillance to the National Health Mission to support other health activities.

*Ethiopia* eliminated polio relatively early, ending transmission of wild polio in 2001, shortly after the first nationwide house-to-house campaign. This was in part the result of a deliberate ‘diagonal’ approach to polio campaigns, in which disease-specific programmes are used to support broader health systems functions,^28 29^ amid a government-led commitment to PHC. The government made a concerted effort to use polio funding to bolster surveillance and cold chain activities that benefited the Health Extension Programme.^3^ However, access to immunisation and other health services in Ethiopia remained uneven and Ethiopia experienced multiple subsequent polio outbreaks. Nonetheless, the numbers of campaigns carried out in Ethiopia have been comparatively modest, between one and two national campaigns per year; in high-risk areas or during active outbreaks, additional subnational campaigns—as high as five annually—have been conducted.^30^

*Nigeria* was one of the last three countries in the world, along with Afghanistan and Pakistan, to harbour wild polio transmission. The entrenched nature of endemic polio in northern Nigeria, an area with low RI coverage, led to an intense focus on polio in the region both nationally and internationally. By the mid-2000s, households across Northern Nigeria were targeted by a polio campaign every other month, a trend that continued for over a decade. Nigeria saw its last case of wild polio in 2016, but Nigeria currently suffers from extensive circulation of vaccine-derived polio.

### Analysis

Before beginning our analysis, we compiled a list of variables identified as mediating the relationship between campaigns and health systems in previous studies.^3 4 31^ These variables fell into five clear themes: FLHWs; supervisory structures; politics and government-community relations; health system quality and responsiveness; and the amount and nature of reliance on mass campaigns.

All the literature and interviews in our dataset had been previously coded. We collated coded material from both studies according to these five themes. Three analysts went through the data, each focusing on material across the two studies collected in a single study country. We authored extensive memos describing each theme over time within each study country,^32^ and engaged in weekly review sessions to discuss findings across countries.

Drawing on this analysis, the team developed a CLD to reflect the interactions between polio campaigns and health systems across the three study settings. Unlike other CLDs built and tested using quantitative methods, we took a strictly qualitative approach to analysis, adhering to qualitative standards for high-quality, inductive theory development.^33^ Each analyst developed an initial CLD independently, to describe the dynamics in the country they had been focusing on; these diagrams were shared among analysts and where differences occurred, they were discussed until we understood them. We then drew on these three, country-specific CLDs to create an overall CLD that included dynamics that were consistent across all study countries. Subsequently, the team iterated over many weeks to ensure the CLD accurately reflected inductive findings from the data, included key concepts, and was interpretable. The CLD was further validated by coauthors in each of the three study countries.

## Results

**Figure 1:**
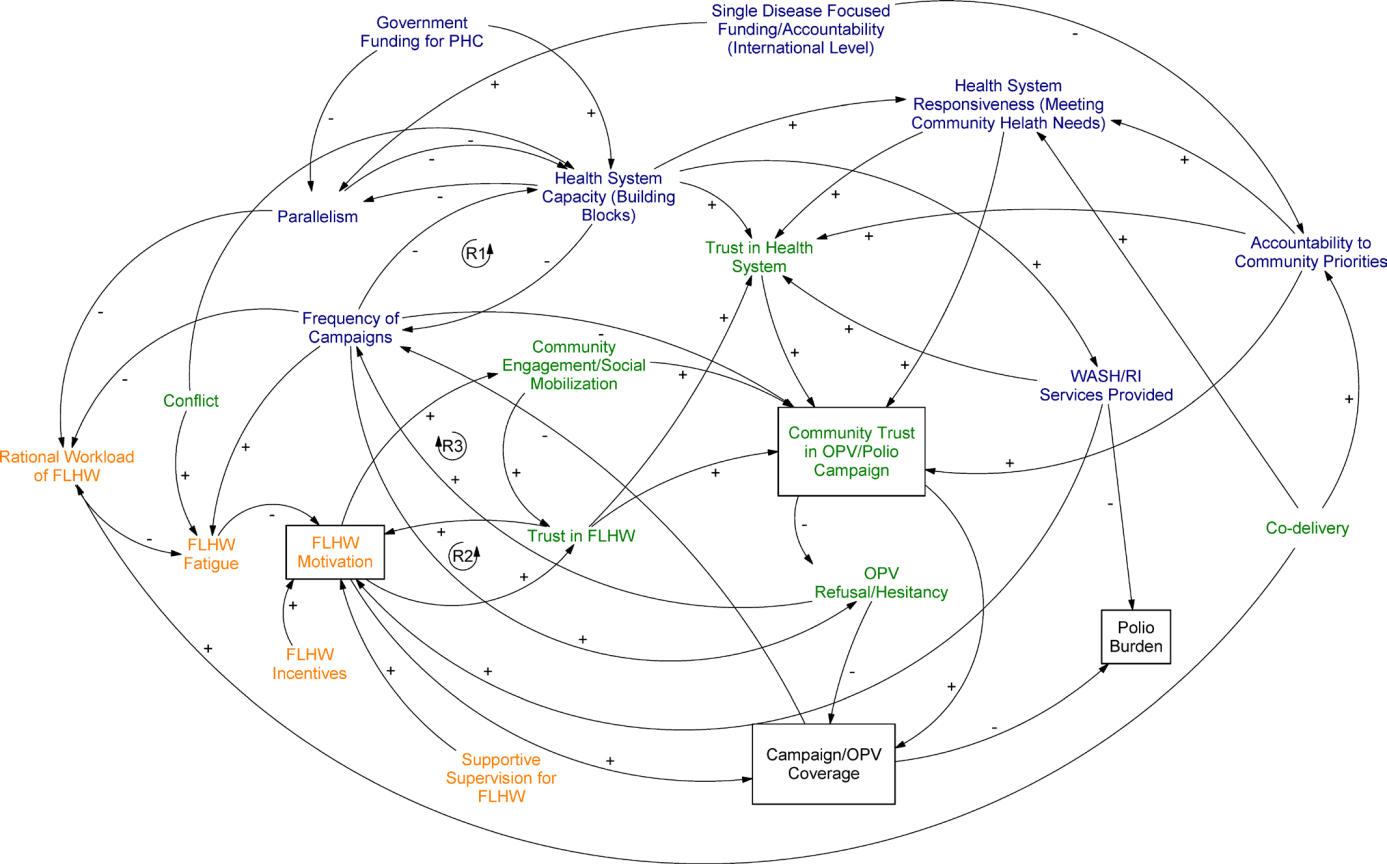
Causal loop diagram (CLD) showing interactions between polio campaigns and health systems across Ethiopia, India and Nigeria.

Our CLD (figure 1) provides insight into several aspects of the interaction between the polio programme and health systems that were not emphasised in previous studies. At the policy/national level, it highlights the path dependency of establishing parallel systems—where the GPEI built parallel structures, it pushed the programme down particular paths of increasing numbers of campaigns, and reduced accountability to local communities. These path dependent outcomes filter down to lower levels of the health system. At the frontline worker level, parallel systems create a cascade of effects that can demotivate workers. At the community level, these cascading effects can lead to vaccine hesitancy.

The CLD shows how campaigns interact with health systems over time to affect *system outcomes*, for example, FLHW motivation, and the effects of those system-level outcomes on *polio program outcomes*, for example, OPV campaign coverage. A positive arrow (+) means that an increase in variable A will lead to an increase in variable B. A negative arrow (−) indicates that an increase in variable A will lead to a decrease in variable B.^14^ An increase in the frequency of polio campaigns, for example, increases (+) health worker fatigue, whereas a rational workload for FLHWs reduces (−) fatigue. We show *simultaneous causation* where variable A can cause variable B, and variable B can also cause variable A at the same or different times. This is depicted as a *feedback loop*. In a reinforcing loop, for example, the link between health worker motivation and trust, the links between the variables move in the same direction, creating an amplifying effect: when health workers are trusted, they are more motivated, leading them to act in ways which further reinforce trust. We highlight *path dependency* by depicting multiple pathways that may exist between one variable and another, for example, the relationship between FLHW motivation and OPV coverage can operate through community engagement, trust in the FLHW, or directly.

Here, we divide the CLD into three sections: the policy level; the FLHW or service delivery level; and the community level. While the relationships described in the CLD hold across all contexts in the study, not all dynamics are salient in each national or subnational context (eg, conflict is not present everywhere).

We focus in the text on several key outcomes of interest (boxed terms in figure 1), particularly the cascading effects that drove frontline worker motivation and vaccine hesitancy over time. For additional information on some key dynamics affecting frontline worker motivation not covered in the main text, see the online supplemental material 1. Terms in the CLD are bolded in-text for ease of reference. In each section, we present the same figure, with the relevant interactions discussed in that section highlighted.

10.1136/bmjgh-2021-006002.supp1 Supplementary data

### Policy level

#### A vertical program meets diverse health systems

At the policy level, the **single disease focus** of the GPEI impacted outcomes through two main pathways: (1) through **parallelism,** that is, the construction of parallel systems to deliver polio vaccination through single-disease campaigns, rather than by improving RI and WASH infrastructure over the long term; and (2) through pushing health systems to focus on global agendas, reducing **accountability to community priorities** and **responsiveness to community needs**.

Strengthening RI has long been a stated goal of the GPEI. Indeed, the 1988 World Health Assembly Resolution for the worldwide eradication of polio called for actors to pursue eradication in a way that would ‘strengthen national immunisation programmes and health infrastructure’.^34^ Still, some senior leadership historically viewed attempts by polio staff to strengthen RI or WASH—both of which reduce polio incidence—as a distraction from the single-minded focus needed to eradicate a disease globally.^3^ A global-level official commented in 2019:

Some people would call them the blinders that polio often has, and others would say that it’s actually the extreme focus that the program has on a very specific goal and target, so either way, it can be phrased positively or negatively…But it was quite difficult…to get the program to be more lateral looking and to understand that, you know, a day spent on strengthening routine immunization isn’t a day lost. (Global official, 2019)

In practice, in our study countries, the GPEI focused heavily on OPV campaigns, leading to mixed impacts on RI systems.^10 35^ In areas where health systems were too weak to support implementation of polio eradication activities at scale and with fidelity, the GPEI created parallel structures—initially designed to be temporary—in an attempt to eradicate polio on a short time frame.^3 18^

The scale of this **parallelism** was dependent on **health system capacity** at both national and subnational levels. In places with relatively strong health systems and substantial **financing for PHC**, polio campaigns were largely implemented though government structures, and polio eradication often provided helpful health system inputs. Strong **WASH and RI** services helped to eliminate polio more easily in these areas.^1 3^

In South India, for example, stronger health systems meant that a few polio campaigns a year could be integrated into existing health system infrastructure. In Ethiopia, polio-funded trainings were used to educate health workers in areas beyond polio vaccination, including deworming and screening for fistula. Polio funds also supported the diagnosis and surveillance of other vaccine-preventable diseases such as measles, rubella, rotavirus, and influenza. A national level official explained in 2019, ‘There was an effort to develop the health system using polio resources.’

#### Parallelism and campaign frequency in weak health systems

In contrast, where health systems were weak and polio persisted, the eradication programme built parallel structures to supervise key campaign functions. In North India in 2011, there were over 100 UNICEF, WHO and CORE group funded staff in a single district, carrying out polio surveillance and social mobilisation, and supervising campaigns. They were doing so because of a ‘shortage of staff’ in the national program, a national level official said in 2019. ‘The cold chain maintenance was not there; supervisory cadres were missing.’ Pushing to eliminate polio on a short timeline, international agencies placed their own polio-specific staff in key districts, rather than engaging in the slow work of strengthening the health system.

The GPEI also made the decision at the international level to increase the frequency of campaigns in polio-endemic areas, to as many as eight times per year in Northern Nigeria and eleven per year in North India. Because campaigns required the labour of government frontline and district health staff as well as polio’s own staff, the polio programme made its most intense demands on health systems in the places where those systems were the weakest.^3 18 35^

**Figure 2:**
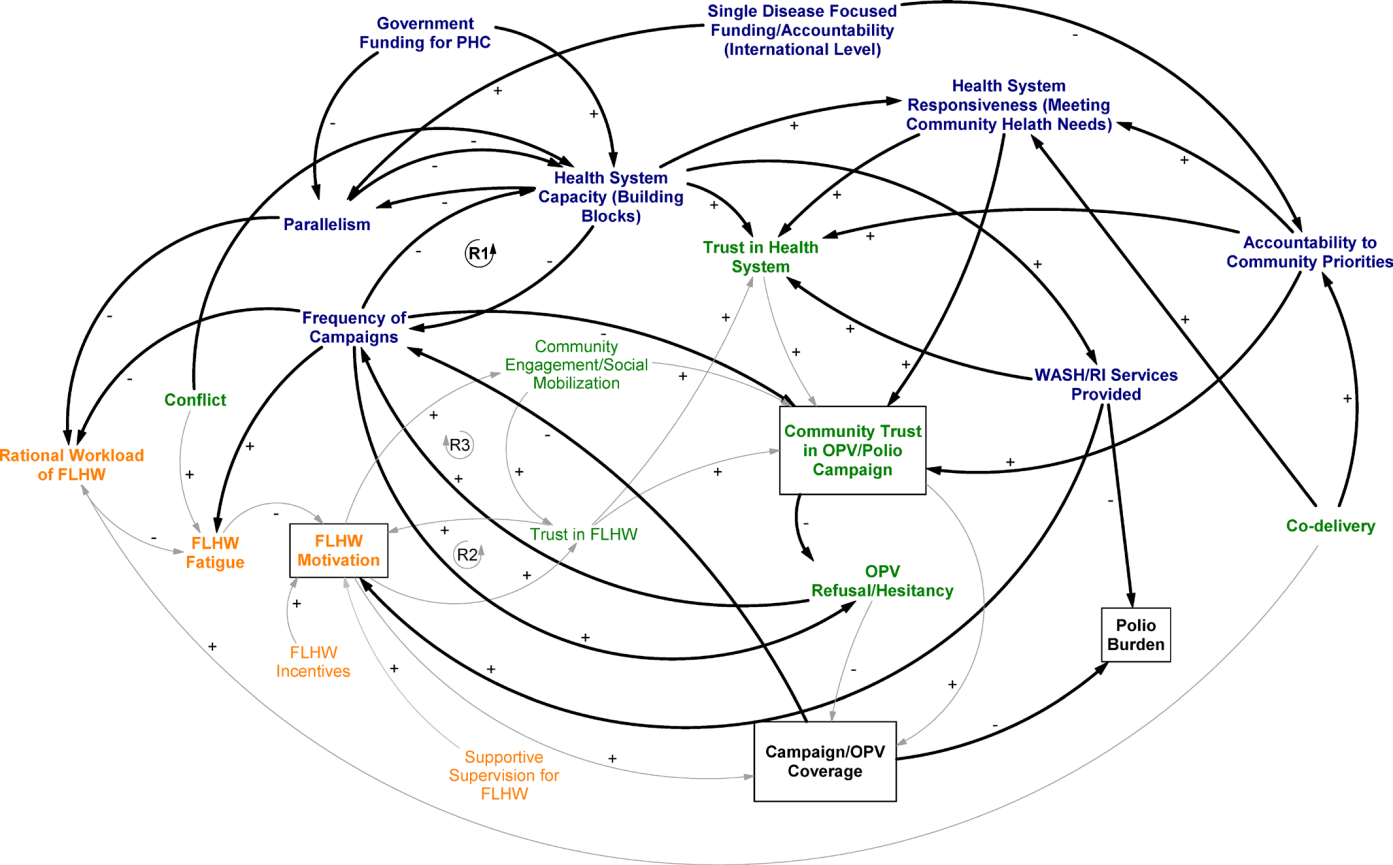
CLD with national health system dynamics highlighted. FLHW, frontline health worker; OPV, oral polio vaccine; PHC, primary health care; RI, routine immunisation.

This relationship is represented in the reinforcing loops between **frequency of campaigns** and **health system capacity** in figure 2. Northern Nigeria is a key example of this dynamic. In a presentation at the National Vaccine Summit in 2012, a meeting designed to shore up support for Northern Nigeria’s struggling RI programme, a national level official wrote,

As we give the final push to finish the job of eradicating polio…we must recognize that routine immunization service delivery in fixed facilities has suffered, and the immunization system has slowly evolved into a campaign-dependent service.

Officials on the ground had similar concerns:

Areas where supplemental immunization [polio campaigns] affect routine immunization are finance and time. This is because most of our energies, most of the meetings we hold are on the supplemental immunization rather than on routine immunization. The time and the energy of most of the health workers are mostly used for supplemental immunization. (District level official, Kano, Nigeria, 2012)

A heavy focus on frequent campaigns was designed to be a temporary measure for meeting a critical health need. As polio eradication efforts dragged on for decades in our study countries, however, these parallel structures became semi-permanent.

### Frontline health worker level

#### Campaign fatigue and FLHW motivation

More than any other actors, respondents said that FLHWs were key to ensuring that vaccinations were delivered and communities engaged. The **motivation** of frontline staff was directly related to **campaign OPV coverage**.^36 37^ In our study sites, FLHWs, who were predominantly women, and ranged from nurses, to community health workers, to community members taught to administer OPV.

Across countries, experienced polio managers said the human element was critical in high campaign coverage, although perhaps not always given sufficient attention. A national-level official in India commented in 2019:

You need tremendous dedication on the part of all the people who are involved in it… There are so many technical challenges that can be solved, but human beings are the most difficult. Fatigue, turnovers, people finding better work to do, misbehavior, not being on time, attitude… So to keep this huge thing running, that’s a tremendous challenge.

Across all settings, even in settings of conflict, frontline workers said that polio program-related **fatigue** was primarily driven by high campaign frequency. In areas with many campaigns, workers repeatedly commented on campaign fatigue; in areas with few campaigns, they rarely mentioned it.

A district level official in Bihar, India, where campaigns were frequent, commented in 2012, ‘Of course, the fatigue level has also increased… for 15–16 years you are doing the same things again and again.’ A national-level official added that in that region, ‘I think that retaining the motivation of human resources despite the fact that they did it every time was very, very, very difficult.’

Frontline workers had existing responsibilities for a range of health initiatives. Polio activities on top of these made the workload difficult for many. In Bihar, India, FLHWs spent at least 77 days per year on polio campaigns alone.^3^

A district official in Ethiopia explained in 2019 that during campaigns, workers ‘start working at 7 am; and in the evening they stay late, up to 8 pm. And, they have to work on Saturdays and Sundays.’ While campaign frequency was relatively low in Ethiopia, Health Extension Workers were expected to perform regular tasks for 16 different packages as outlined by the Ministry of Health. Many of these tasks, including other vaccinations for which the district was responsible, were not amenable to campaign delivery.

Frontline Ministry of Health workers were often accountable to multiple supervisors in different parallel programmes, with different workflows and reporting requirements. Their workflow often made little logical sense. In parts of North India in 2012, the same workers visited the same children multiple times in a single month with completely separate campaigns for polio vaccination, measles vaccination, and vitamin A.

Such unnecessary duplication of effort, along with different supervisory and remuneration structures for every vertical programme, was frustrating for frontline staff. Lack of convergence, as one official in Bihar, India, described it, meant that programmes for polio, kala azar, leprosy, HIV/AIDS, and others were financed, organised, and implemented independently of one another. While the resources each vertical programme brought had impacts on their specific diseases of interest, this official commented, they pulled workers in many different directions, and left core functions like RI without support.

In less parallel systems, for example in Southern Nigeria and South India, government staff planned and monitored both campaigns and routine services, usually leading to more **rational workloads** for FLHWs. **Co-delivery** also helped to streamline workloads and reduce fatigue among frontline workers in all three study countries. In fact, because it improved workflows so significantly, co-delivery often occurred on an ad-hoc, informal basis even when it was not planned at the national level. In Ethiopia,

polio health workers became so familiar with communities and households while making their rounds [during campaigns], after some time they also started delivering newborn and maternal health interventions, participating in identification of children with cleft lip, and the like…they are already working by integration. (District level official, Ethiopia, 2019)

In India,

…what was interesting is by the time the program almost came to an end, when we were near to eradication, these Community Mobilization Coordinators [polio frontline communications workers] were covering all the health programs because ‘ye bhi karo woh bhi karo kar kar ke tum to jaa hi rahe ho’ [Do this, and do that as well, because you are already going there]. (National level official, India, 2019)

While these informal solutions were often effective in practice, many respondents said that an integrated national policy, thinking through frontline workflows, should have been embedded in planning from the start.

Importantly, parallelism’s impacts on fatigue did not occur right away—these effects were slow burning. Strategies like frequent campaigns and parallel systems were often quite effective in the short term, but over years, especially when combined with poor **incentives** and lack of **supportive supervision**, drove worker fatigue in powerful and at times corrosive ways. ‘Polio, polio, polio,’ a frontline worker in North India complained. ‘When will anyone pay attention to anything else?’ Some Indian researchers commented:

To start with, the motivation level in the community and among the volunteers and health workers was very high…. However, in recent years, it has been observed that because of repetition of [polio] activities year after year, community participation is on the decline and there is fatigue among the beneficiaries. The motivation level among the volunteers and health workers is at its lowest ebb.^38^

#### Motivated FLHWs build community trust

Highly motivated workers contributed to more effective and sustained **community engagement**. A national-level Indian official commented in 2019 that high quality community engagement was part of a suite of tasks that workers needed to tackle on a ‘street by street basis.’ He explained:

In getting them as local women to engage mothers in courtyards, in households, at the mosque, to talk about why their kids should be immunized. And then, recording the precise records of every single child by name in a village, so that when the vaccinators would come, they could say at the end of the day who did they miss. So, we use that level of precision… in order to be successful.

**Figure 3:**
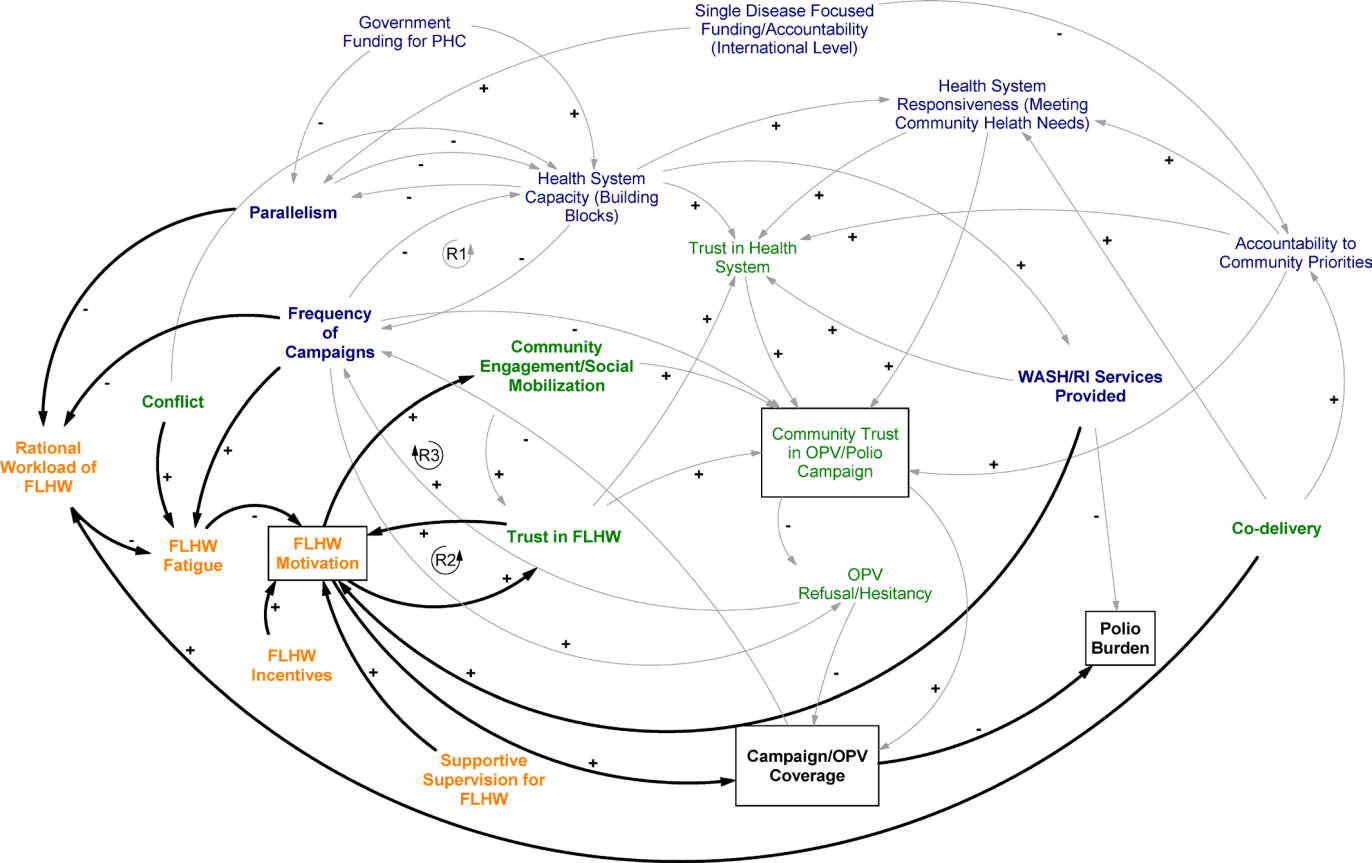
Causal loop diagram with frontline health worker (FLHW) dynamics highlighted. OPV, oral polio vaccine; PHC, primary health care; RI, routine immunisation.

Across place and time, effective community engagement, carried out by motivated workers, led to greater levels of community **trust** in the workers themselves and, by extension, the vaccination campaign. This dynamic is the third reinforcing loop in figure 3: when health workers were trusted, they became even more motivated.

In both strong and weak health systems, FLHWs bore the responsibility of sustaining, or winning back, community trust.

### Community level

**Figure 4:**
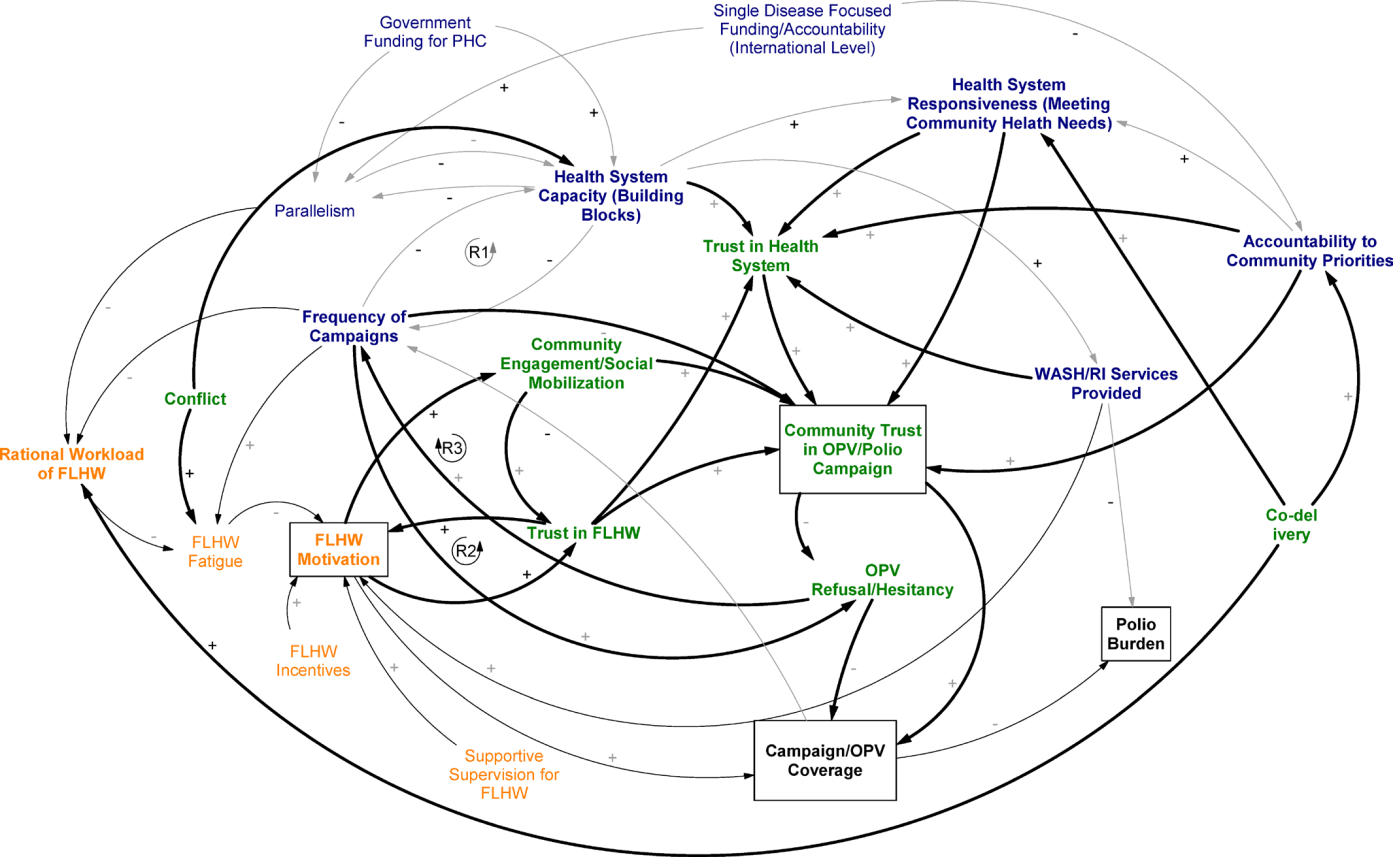
Causal loop diagram with community dynamics highlighted.

Polio vaccine hesitancy in our study countries has sometimes been conceptualised as arising from factors internal to the community itself.^39–41^ However, as figure 4 demonstrates, vaccine hesitancy is also integrally tied to health systems dynamics.

#### Quality of the broader health system: key to trust in campaigns

Trust in polio campaigns was powerfully shaped by communities’ **trust in the health system** more broadly. In South India, for example, community members expressed enthusiasm for the health services accessible to them, and were generally happy with polio campaigns; however, in North India, the Indian Academy of Pediatrics noted:

RI coverages are suboptimal and underserved communities have a tendency to suspect the intentions of the specific focus on polio… while the community demands measles and DPT vaccination.^42^

In parts of North India and Northern Nigeria during the years covered by our studies, facilities were often closed, or without essential staff or medicines. A report on the district that harboured polio cases in India longer than any other noted,

The community does not have enough confidence in the government facilities since the personnel are not always available especially Lady MOs [female doctors] and also adequate infrastructure, equipment and drugs.^43^

When health systems were generally not **accountable to community priorities**, people questioned why polio vaccine was delivered to their doorstep.^13 24^ A district health official in Northern Nigeria commented in 2012:

Sincerely speaking, the public is so suspicious about the campaign on polio. People need malaria tablets more than polio vaccine… Some people complained that when fuel prices were increased nothing was done to console the poor, but when polio was rejected by the poor, the government and the community elders were used to persuade people to accept it…People are saying that malaria tablets should be provided rather than polio vaccines, if the government really wants to help them. 55% of the public are not satisfied with the vaccine.

In some cases, lack of **accountability to community priorities** led to **OPV refusals** that were strategic: that is, people trusted the polio vaccine, but they refused it in an attempt to get the government to provide them with services they needed. These so-called ‘demand refusals’ were largely confined to areas with frequent campaigns.

One example is North India, where parents refused OPV in an attempt to draw attention to the food supplementation that was due their families. A FLHW explained in 2012, ‘people say that I did not get the rice and pulses, so I will not give the polio drop to my kids.’

Such frustration over the focus on polio was especially apparent in areas with **frequent campaigns**. Dissatisfaction did not arise right away, but could become intense over years of repeated polio rounds. ‘Ahh some say it is too much, too many rounds, they are tired,’ a frontline worker in Nigeria said in 2012. A district-level official in northern Nigeria explained in 2012:

In a year you have like 10 campaigns, which leads to dissatisfaction and people saying there are misplaced priorities…. It is not that people are rejecting polio [vaccine], not even those that are refusing or denying vaccination. They do that due to the negligence from the system of some high priority. Sometimes you will see a caregiver telling you, “I have taken my child to the hospital but there were no drugs, so why are you now delivering polio vaccine to my house? I had to buy the drugs prescribed for my child somewhere else.” Leaders should intensify effort on key areas of the PHC so that we can achieve our aim of polio eradication.

A frontline worker in the same district commented:

The members of the communities always complain about the constant house-to-house visits, mainly only for the polio activities. They normally express their uncertainly about government mission of polio eradication alone; after all, there are so many diseases like measles and so on that need to be eradicated from the community.

#### Co-delivery to address community hesitancy

In all of our study countries, however, community hesitancy of this nature was mitigated through increased **health system responsiveness to community needs**. In part in response to community demands, and in part because they felt a moral imperative, health officials began to use GPEI resources and implementation mechanisms for broader health services delivery. **Co-delivery** of health interventions encompassed a suite of ways to increase responsiveness to community needs, even while operating within the confines of a parallel vertical programme. In Ethiopia and Nigeria, OPV was increasingly delivered alongside Vitamin A, insecticide-treated nets (ITNs), and deworming tablets, and CORE group volunteers engaged broadly in child health education.^44^

India’s 107 Block Plan, developed in 2009, focused on RI, sanitation practices, breastfeeding rates, and reducing diarrheal disease. This convergent action contributed to the elimination of polio transmission in India.^3^ An Indian official explained in 2019:

Our training was only polio initially, our communication package was only for polio. [Frontline workers said] every time I am talking to mother about polio, her child is having fever, child is having diarrhea, child is having other problem. So, we added other diseases in [the communications] package like diarrhea control, ORS distribution, so then the community got some trust: these people are not only interested in polio but also in my child’s health.

While using polio structures as a platform for other services made the health system overall increasingly reliant on the polio programme, respondents described a palpable, positive difference in the way campaigns were received as co-delivery increased.

This [last campaign] was also different in a way that we tried to integrate different interventions, like we integrated the measles [vaccine]… nutritional screening, and vitamin A, and also de-worming. All these were integrated and this was a campaign with a high coverage because—I would say—different from the other campaigns. The Ministry [of Health] took the ownership for this particular campaign. Previously, more or less the campaigns were kind of organized and led by partners like WHO and UNICEF. (National level official, Ethiopia, 2012)

## Discussion

Applying a CAS approach to the relationships between polio campaigns and broader health systems highlights some implementation pathways that are less apparent in more traditional, static approaches to health systems analysis. CLDs allow us to visualise feedback loops: for example, the simultaneous cause-and-effect relationships between FLHW motivation and community trust. Both positive and negative relationships are possible within the same loop. Such analyses also allow exploration of how the nature of these relationships changes over time. Feedback loops and change over time are properties of most health systems, as well as efforts to strengthen health systems or to deliver vertical programmes. Thus, the CLD confers an analytical advantage in that it allows us to examine these relationships as closer to how they occur under real-world conditions. In this paper, highlighting the interconnectedness of the health system, its subsystems, and campaigns helped illuminate path-dependent unintended consequences that affect campaign effectiveness over time.

The CLD approach is increasingly being used to analyse a variety of health systems dynamics, from performance-based financing to essential drugs policies to the scale-up of health services.^20 45 46^ The CLD approach could usefully be applied to analyse other vertical programmes (eg, HIV, COVID-19, malaria) that interact with the health system, even if the dynamics at play are different than the ones explored here. Future work might also usefully explore applications of the CLD to other key issues in this arena, such as planning for donor transitions, and predicting unintended consequences prior to introducing new global programmes.

In the case of polio, the dynamic interactions between mass vaccination campaigns and health systems caused polio campaigns to become less effective over time in areas with weak primary health systems. These interactions developed over years, through cascading effects from the policy, to service delivery, to the community level of the system. Where health systems were insufficiently responsive to community needs and priorities, pursuing frequent campaigns through parallel systems led, over time, to a loss of FLHW motivation and an increase in vaccine hesitancy. Co-delivery of interventions helped to mitigate the negative effects of parallelism, though an overreliance on externally funded vertical programmes to deliver core health services may threaten sustainability of these services over time.

Over 25 years ago, the Taylor Commission expressed concern over a nascent problem: ‘a fatigue effect on communities with the insistent emphasis placed on vaccinations’.^1^ Polio implementers have indeed been confronted by these challenges over the course of a long eradication effort, most acutely in the final frontiers of polio eradication. Nigeria is a clear example of this,^40^ as are Pakistan and Afghanistan, which in recent years have experienced an increase in demand refusals and risks for FLHWs.^47–49^

Eradication programmes are particularly susceptible to these issues. The polio programme has adapted to on-the-ground implementation challenges in every region of the world but has often done so by working around the health system. Cassandra White notes that ‘a sustainable strategy must incorporate the voices and knowledge of people affected by the disease and of healthcare professionals involved’ even if that means releasing the allure of disease eradication.^50^ While COVID-19 does not have an eradication agenda, such issues are likely to be central in the era of COVID-19 vaccination, particularly given the potential for frequent campaigns over a prolonged period, as well as the significant political attention to this issue.

Vaccine hesitancy is of course a key issue in the COVID-19 era. Already, researchers have, for example, provided psychological explanations for COVID-19 vaccine hesitancy, and pointed to the importance of the historical foundations of trust.^51 52^ An understanding of the past is of course important. This analysis shows, however, that the *current* design of mass vaccination programmes is also critical, even if it is not initially a reason for vaccine hesitancy. A focus on a single disease in a population underserved by the health system can lead to slow-burn opposition to a vaccine that takes time to arise.

A key dynamic here is power: who has the ability to set agendas for communities? Where power is applied to push through a specific vaccination agenda when frontline workers and communities have different agendas, resistance *will* arise. Frontline worker resistance can look like shoddy work or ‘fatigue’—but resistance is a better frame for understanding it.^37^ We have glossed community resistance as ‘hesitancy’ in this paper, but in fact, demand refusals are not really hesitancy: they are organised resistance to the application of power.

While we endeavoured in both studies to include a diverse, representative group of respondents, our methods did have limitations. Our analysis may have benefited from additional perspectives, including from caregivers, and from additional health managers outside of the polio programme. Nonetheless, our material points to a few productive ways forward for mass vaccination programmes.

First, programmes should limit reliance on single-disease campaigns. Ideally, single-disease initiatives would be fully integrated into national health systems. But at a minimum, co-delivery of interventions can minimise negative health systems outcomes, even in a parallel system.

Second, FLHW burden should be carefully considered across programmes. Reducing campaign fatigue can positively impact FLHW motivation. Third, if a vertical programme targets an issue that is not a community’s top priority, it should be considered carefully, and other needs assessments conducted prior to developing strategies for high-risk areas. At a minimum, an intervention that is not addressing a community priority should be paired with one that is.

Finally, to design and implement effective campaigns, policy-makers must consider campaigns within the context of known health systems dynamics: path dependency and interconnectedness. On-the-ground implementation of campaigns cannot be separated from broader contextual forces. Implementation strategies to improve fidelity or efficiency of campaigns are pivotal for ensuring effective delivery in the short to medium terms, but to be effective over an extended time horizon, campaign strategies must be developed in a way that makes sense for those delivering and receiving campaign interventions.

## Data Availability

Data are available upon request. Data will be made available upon reasonable request where sufficient deidentification of interview data transcripts can be made. Data requests can be directed to Svea Closser at sclosser@jhu.edu and Olakunle Alonge at oalonge1@jhu.edu.
