# Supplementary material for: 30 years of polio campaigns in Ethiopia, India and Nigeria: the impacts of campaign design on vaccine hesitancy and health worker motivation
Source: BMJ Glob Health. 2021 Aug 3;6(8):e006002. doi: 10.1136/bmjgh-2021-006002 (PMC8336205; doi:10.1136/bmjgh-2021-006002)
Supplement: Supplementary data [file bmjgh-2021-006002supp001.pdf]

Supplemental Material
Additional descriptions of variables included in the Causal Loop Diagram (CLD)

Our causal loop diagram (CLD) (Figure 1) describes interactions between polio campaigns and health systems across Ethiopia, India, and Nigeria and how those interactions affect both *system outcomes* and *polio program outcomes* over time, following standard CLD notation. In the main text, we have highlighted variables and their interactions that are particularly salient to the outcomes of interest in our analysis, or where current research is limited. We have included below additional data on variables which are well covered in the literature (e.g., conflict, frontline health worker (FLHW) incentives, and supportive supervision for FLHWs), but which are corroborated by our data. Additional information on the qualitative data underlying this analysis is available upon request.

CLD Notation
A positive arrow (+) means that an increase in variable A will lead to an increase in variable B. A negative arrow (-) indicates that change occurs in the *opposite direction*, or that an increase in variable A will lead to a decrease in variable B (14). An increase in the frequency of polio campaigns, for example, increases (+) health worker fatigue, whereas a rational workload for frontline health workers reduces (-) fatigue. We show *simultaneous causation* where variable A can cause variable B, and variable B can also cause variable A at the same or different times. This is depicted as a *feedback loop.* In a reinforcing loop, for example the link between health worker motivation and trust, the links between the variables move in the same direction, creating an amplifying effect: when health workers are trusted, they are more motivated, leading them to act in ways which further reinforce trust. We highlight *path dependency* by depicting multiple pathways that may exist between one variable and another, e.g., the relationship between FLHW motivation and OPV coverage can operate through community engagement, trust in the FLHW, or directly.

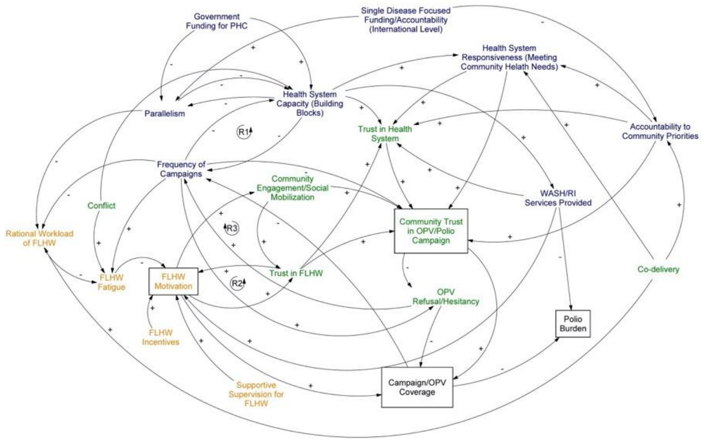

Figure 1. Causal loop diagram (CLD) reflecting interactions between polio campaigns and health systems across Ethiopia, India, and Nigeria

Conflict

Although not a major factor in India, **conflict** had a significant effect on the fatigue and workload of frontline workers in insecure parts of Nigeria and Ethiopia. This was a particular issue in Northern Nigeria, where frontline health workers feared for their safety in the context of Boko Haram kidnappings, civil unrest, and lethal attacks on polio workers (Smith 2013).

> *The security situation in Nigeria is far worse than it was a year ago. We sympathise with the difficulties that this may present to the programme. The programme must strive for better performance to mitigate these challenges. As we discuss later, country programmes need to be able to deal with insecurity if they have a genuine ambition of stopping polio transmission.* (Global Polio Eradication Independent Monitoring Board, 4th report, February 2012)

Given related population movement, conflict also challenged surveillance efforts and made it challenging to assess campaign effectiveness, a fact that was noted among both Ethiopian and Nigerian respondents:

> *The insecurities, especially for the past 3 or 4 years, had an effect. Because of this, knowing the status of community-based intervention becomes difficult; it was hard to be certain whether there are cases or not. So we could say the instability has an effect.* (Ethiopia, 2019)

> *Conflict means populations are moving consistently, trying to avoid things, so they miss vaccinations, you can't even reach them to vaccinate them, new children are born, and they don't get vaccinated because you can't go there. So, the more you don't do that, they more they are going to be prone to be more susceptible, because they are not vaccinated, so yes, conflict is a major, major, major factor and has really affected the Nigerian program a lot.* (Nigeria, 2019)

FLHW Incentives

One key determinant of frontline health worker motivation was **incentives**—specifically, adequate and timely remuneration. At times in parts of India and Nigeria, frontline worker pay, which was low across our study countries, was late. Regardless of whether the pay was for the frontline workers' routine tasks or for their polio tasks, late pay negatively affected motivation. For example, an ASHA in North India commented angrily,

> *It's been three years, that we did not receive anything [for our regular ASHA salary]. I have my stomach to feed, and three children, who go to private school. I am looking for another job so that I will be able to feed my family.* (India, 2012)

In Ethiopia, a frontline worker commented bitterly on the disparity between their polio incentives and their supervisors' salaries:

> *Many are benefiting from [the polio program] without effort, but the one suffering for it is different. Every administrator is included [in the payment], I don't know the reason… It has reached the level of hatred [for me]. The payment is not fair. Administrators get paid better than the one who vaccinates through a house-to-house visit.* (Ethiopia, 2019)

The amount of pay was an issue across our study countries, and it was a particularly acute problem when the expenses involved in reaching remote areas were significant. A policymaker in Nigeria said that the pay was "really found to be below minimum and it affects performance." A frontline worker in Nigeria commented, "If I have to spend N500 and you give me N100, I might not reach that place."

Neel AH, *et al. BMJ Global Health* 2021; 6:e006002. doi: 10.1136/bmjgh-2021-006002

Supportive Supervision for FLHW

Supportive supervision, including opportunities for training and advancement within the health system, was also critical for frontline worker motivation. Frontline workers in Nigeria said that punitive structures and poor supervision drove them to falsify numbers of children vaccinated to make their work look good on paper. Rewards for quality work were also important—without them, workers lost motivation. A female frontline worker in Ethiopia commented in 2019, “The lack of incentives, working at one place without a promotion, and being posted far from families…brings on a careless behavior in workers.”

Over time supportive supervision and other accountability mechanisms became a focus within the polio eradication initiative. This led to improvements in campaign effectiveness, but as some noted, supervision was often lacking for other health services, including routine immunization, and was the level of supervision for polio tasks was not always extended or sustained beyond the polio program. As one official in Indai noted,

> *In the Polio Programme the main thing was the supervisors were given an emphasis as to how to supervise, now that has really fallen apart. I think that was something taken up [in a]big way for polio eradication. Now, the skill of a supervisor is not given that much importance.* (India, 2019)

Impacts within the CLD

Across place and time, effective community engagement, carried out by motivated workers, led to greater levels of community **trust** in the workers themselves and the entire campaign. In Northern Nigeria, social mobilization and community engagement activities carried out by frontline workers to engage youth, women, and marginalized groups led to more awareness, trust, and better coverage in communities. High levels of trust built over several years of campaigns in India led to a relatively smooth switch from oral to injectable polio vaccination:

> *People were thinking that when this injectable was introduced, we were telling people over the last 10 years that oral is the best suddenly we started saying that injectable is the best, so we thought that it would be a challenge for us to again convince the community, but touch would our engagement with the community and the confidence the community showed on us, we didn’t find any issue in the introduction of this injectable. So, it shows your work, your reach and I think the confidence we won, that paved the way for the smooth transition and introduction of new vaccine.* (India, 2019)

Across our study settings, even in settings of conflict, frontline health workers stated that fatigue was primarily driven by high campaign frequency. Still, these other variables played, and continue to play, a significant role in driving frontline health worker performance. Given their central role in delivering effective campaigns and enabling community trust, these variables continue to warrant attention from policymakers and public health implementers.

Neel AH, *et al. BMJ Global Health* 2021; 6:e006002. doi: 10.1136/bmjgh-2021-006002
